# Telomere attrition in heart failure: a flow-FISH longitudinal analysis of circulating monocytes

**DOI:** 10.1186/s12967-018-1412-z

**Published:** 2018-02-20

**Authors:** Iris Teubel, Elena Elchinova, Santiago Roura, Marco A. Fernández, Carolina Gálvez-Montón, Pedro Moliner, Marta de Antonio, Josep Lupón, Antoni Bayés-Genís

**Affiliations:** 1 Flow Cytometry Facility, Germans Trias i Pujol Health Science Research Institute, Badalona, Spain; 2 Cardiology Service, Germans Trias i Pujol University Hospital, Carretera del Canyet s/n, 08916 Badalona, Spain; 3 Department of Medicine, Universitat Autònoma de Barcelona, Barcelona, Spain; 4 ICREC Research Program, Germans Trias i Pujol Health Science Research Institute, Badalona, Spain; 5 Center of Regenerative Medicinein Barcelona, Barcelona, Spain; 6 CIBERCV, Instituto de Salud Carlos III, Madrid, Spain

**Keywords:** Heart failure, Monocyte subsets, Telomere attrition, Telomere length

## Abstract

**Background:**

Cross-sectional investigations report shorter telomeres in patients with heart failure (HF); however, no studies describe telomere length (TL) trajectory and its relationship with HF progression. Here we aimed to investigate telomere shortening over time and its relationship to outcomes.

**Methods:**

Our study cohort included 101 ambulatory patients with HF. Blood samples were collected at baseline (n = 101) and at the 1-year follow-up (n = 54). Using flow-FISH analysis of circulating monocytes, we simultaneously measured three monocyte subsets—classical (CD14^++^CD16^−^), intermediate (CD14^++^CD16^+^), and nonclassical (CD14^+^CD16^++^)—and their respective TLs based on FITC-labeled PNA probe hybridization. The primary endpoints were all-cause death and the composite of all-cause death or HF-related hospitalization, assessed at 2.3 ± 0.6 years. All statistical analyses were executed by using the SPSS 15.0 software, and included Student’s t test and ANOVA with post hoc Scheffe analysis, Pearson or Spearman rho correlation and univariate Cox regression when applicable.

**Results:**

We found high correlations between TL values of different monocyte subsets: CD14^++^CD16^+^ vs. CD14^++^CD16^−^, R = 0.95, p < 0.001; CD14^++^CD16^+^ vs. CD14^+^CD16^++^, R = 0.90, p < 0.001; and CD14^++^CD16^−^ vs. CD14^+^CD16^++^, R = 0.89, p < 0.001. Mean monocyte TL exhibited significant attrition from baseline to the 1-year follow-up (11.1 ± 3.3 vs. 8.3 ± 2.1, p < 0.001). TL did not significantly differ between monocyte subsets at either sampling time-point (all p values > 0.1). Cox regression analyses did not indicate that TL or ΔTL was associated with all-cause death or the composite endpoint.

**Conclusions:**

Overall, this longitudinal study demonstrated a ~ 22% reduction of TL in monocytes from ambulatory patients with HF within 1 year. TL and ΔTL were not related to outcomes over long-term follow-up.

**Electronic supplementary material:**

The online version of this article (10.1186/s12967-018-1412-z) contains supplementary material, which is available to authorized users.

## Background

Heart failure (HF) has become an epidemic, imposing substantial health, social, and economic burdens. In developed countries, HF affects 1–2% of the adult population, with a prevalence of ≥ 10% among those ≥ 70 years of age [1]. It has been suggested that HF is a disease of accelerated aging, and telomere length (TL) is proposed as a biomarker of aging [2]. Telomeres are specialized and evolutionarily conserved tandem repeats (5′-TTAGGG-3′ in humans) located at the end of chromosomes. They serve as protective caps that prevent the DNA damage-repair system from accidentally identifying chromosomal ends as DNA double strands [3, 4]. Preliminary data from cross-sectional studies reveal that persons with HF have shorter telomeres than healthy age- and gender-balanced controls, based on analyses of circulating leukocytes using a conventional quantitative polymerase chain reaction (qPCR)-based method [5]. However, no longitudinal studies have investigated telomere attrition among patients with chronic HF.

TL can also be efficiently assessed in monocytes, a heterogeneous population of effector cells that play key roles in maintaining and restoring tissue integrity [6]. Using flow cytometry, circulating monocytes can be categorized into three distinct subsets based on differential expression levels of the surface markers CD14 and CD16: classic, CD14^++^CD16^−^; intermediate, CD14^++^CD16^+^; and non-classic, CD14^+^CD16^++^ [7]. We recently reported TL assessment in monocyte subsets using a novel standardized analytical protocol based on simultaneous multicolor flow cytometry-fluorescence in situ hybridization (flow-FISH) [8].

In the present article, we report a longitudinal study in which we used the novel flow-FISH technique to explore the dynamics of telomere attrition in circulating monocytes within a cohort of ambulatory HF patients.

## Methods

### Study population

Our study cohort included 101 ambulatory patients who attended a multidisciplinary HF unit from January 15th 2014 to May 6th 2015 (Table 1). The referral inclusion criteria are described elsewhere [9, 10]. All patients made follow-up visits at regular predefined intervals, and additional visits when required in cases of decompensation. The regular visitation schedule included a minimum of quarterly visits with nurses; biannual visits with physicians; and elective visits with geriatricians, psychiatrists, nephrologists, and rehabilitation physicians. Upon missing a regular visit, patients were contacted by telephone.

The primary endpoints were all-cause death and the composite of all-cause death or HF-related hospitalization. Fatal events were identified from electronic clinical records, and by contacting the patients’ relatives when necessary. When verification was required, data were compared with records stored in the databases of the Catalan and Spanish health systems. Events were adjudicated by two of the authors (EE and JL), and by clinical and research nurses.

Each subject gave their written informed consent prior to participation. The study protocol was approved by the Clinical Research Ethics Committee of our institution, was designed in accordance with the principles outlined in the 2013 revision of the Declaration of Helsinki of 1975 [11].

### Blood extraction and processing

Blood samples of ~ 3 ml were collected into EDTA tubes via standard forearm venipuncture performed between 9:00 a.m. and 11:00 a.m., and were processed within 4 h after collection. Samples were collected at two time-points: at baseline (n = 101) and at the 1-year follow-up (n = 54) (Additional file 1: Table S1). Samples from the 1-year follow-up were unavailable due to death (7 patients), technical issues (10 patients), or patient’s unwillingness to repeat sampling (30 patients). Samples and data from patients included in this study were processed and collected by the IGTP-HUGTP Biobank integrated in the Spanish National Biobanks Network of Instituto de Salud Carlos III (PT13/0010/0009) and Tumour Bank Network of Catalonia. All laboratory measurements were performed by staff blinded to the patients’ clinical characteristics.

### Flow–FISH

Blood samples were first lysed by a 10-min incubation with PharmLyse solution (BD Bioscience, San Diego, CA, USA), and then the cell concentration was measured by flow cytometry using Perfect-Count beads (Cytognos, Salamanca, Spain). In a 15-min incubation at room temperature (RT), 1 × 10^6^ cells were stained with titrated amounts of the following antibodies: CD86-BV605, CD14-BV785 (Biolegend, San Diego, CA, USA), CD16-BV421, and CD15-AlexaFluor647 (BD Biosciences). Next, these cells were fixed with 6 mM bis(sulfosuccinimidyl)suberate (Sigma-Aldrich Química SL, Madrid, Spain) for 30 min at 2–8 °C. The reaction was quenched using 1 M Tris buffer (pH 8.0) for 15 min at RT. Then the residual red blood cells were removed by incubation with FACS lysing solution (BD Biosciences) for 7 min at RT.

FISH was performed using the Telomere PNA kit (Dako, Glostrup, Denmark) following the manufacturer’s instructions. The human 4-year old Caucasian female acute lymphoblastic leukemia 1301 cell line from the Health Protection Agency Culture Collections (HPACC) was used with each sample as an internal control. The 1301 cell line was previously cultured according to HPACC recommendations in RPMI 1640 (Gibco, Life Technologies, Grand Island, NY) with 10% fetal bovine serum (Sigma-Aldrich Química SL), penicillin, streptomycin, and glutamine (Gibco, Life Technologies). After purchase of the 1301 cell line, subsequent cells were obtained from four passages after reaching a maximum of 1 × 10^6^ cells viable cells/ml in culture; cells with the same passage number were aliquoted in large numbers and stored at − 196 °C until use. Samples were acquired by flow cytometry, with up to 10,000 monocytes collected per sample. We performed correction for DNA ploidy of the blood sample vs. the internal control as previously described [12].

All samples were acquired on a Fortessa SORP flow cytometer (BD Biosciences) equipped with four lasers (100-mW 488 nm, 150 mW 532 nm, 50 mW 405 nm, and 100 mW 640 nm) using sample acquisition software FACSDiva v6.2 (BD Biosciences) and analyzed with FlowJo vX (Tree Star, Inc, Ashland, OR). We performed routine daily quality control tests with Cytometer Setup & Tracking Beads (BD Biosciences) in accordance with the manufacturer’s instructions. Daily QC control of 6-peak Rainbow Calibration Particles (BD Biosciences) was used for Flow-FISH MFI standardization to reach initial target MFI values. We initially gated for G0/G1 cells of both leukocyte subsets and 1301 cells based on DNA content, and then by scatter properties. Monocytes were sequentially identified using a CD86 vs. CD16 plot, followed by a CD15 vs. CD16 plot to gate out neutrophils. Next, the gated monocytes were analyzed for CD14 and CD16 expression. Clumped cells were excluded using a plot of propidium iodide (PI) area vs. PI width. Finally, each subset of monocytes and internal control cells was displayed on a plot comprising the FITC-labeled PNA probe on PI (B695-A) vs. the PNA probe (B515-A), and the median fluorescence intensity (MFI) of the PNA probe was measured.

The relative TL value for each monocyte subset was calculated as the ratio between the MFI of each subset and the MFI of the control cells. Corrections were made for the DNA index of G0/G1 cells, as previously described [13].

### Statistical analysis

Categorical variables are expressed as percentages. For continuous variables, data distributions were assessed using normal Q–Q plots, and data are expressed as mean and (SD) for normally distributed data, or as median and (quartiles Q1–Q3) for non-normally distributed data. Between-group differences were assessed using Student’s t test and ANOVA with post hoc Scheffe analysis. To assess correlations among the TL of the different monocyte subsets, and between the mean TL of all monocytes and clinical variables, we used the Pearson or Spearman rho correlation test, as appropriate. Comparison between mean TL between baseline and 1-year samples were performed with t test for paired data.

We additionally performed univariate Cox regression analyses with all-cause death and the composite endpoint as the dependent variables, and with the mean TL for monocytes as a whole and for each monocyte subset as the independent variables. In the subgroup of patients for whom a 1-year follow-up blood sample was available, we also assessed the relative TL change using the formula [TL at 1 year –baseline TL]/baseline TL) × 100. Statistical analyses were performed using SPSS 15.0 (SPSS Inc., Chicago, IL, USA). A two-sided p value of < 0.05 was considered significant.

## Results

Table 1 shows the clinical characteristics of the studied population. In general, the patients were middle-aged and predominantly male, showed an ischemic etiology, and were NYHA functional class II or III and treated following contemporary guidelines.

**Table 1:** Baseline characteristics of the study participants

	n = 101
Age (years)	65.6 ± 11.3
Male sex	76 (75.2%)
Etiology	
Ischemic heart disease	46 (45.5%)
Dilated cardiomyopathy	18 (17.8%)
Hypertensive cardiomyopathy	8 (7.9%)
Alcoholic cardiomyopathy	7 (6.9%)
Valvular disease	8 (7.9%)
Hypertrophic cardiomyopathy	4 (4.0%)
Other	10 (9.9%)
HF duration in months	38.7 (12.7–77.4)
LVEF	41.8% ± 12.1
NYHA functional class	
I	9 (8.9%)
II	70 (69.3%)
III	22 (21.8%)
Co-morbidities	
Hypertension	74 (73.3%)
Diabetes mellitus	50 (49.5%)
Renal failure^a^	48 (47.5%)
Anemia^b^	41 (40.6%)
Atrial fibrillation/flutter	42 (41.6%)
Obesity	31 (30.7%)
Smoker	
Current	4 (4.0%)
Past	66 (65.3%)
Treatments	
ACEI/ARB	89 (88.1%)
Beta-blockers	93 (92.1%)
MRA	63 (62.4%)
Loop diuretics	81 (80.2%)
Digoxin	21 (20.8%)
Ivabradine	19 (18.8%)
Statins	86 (85.1%)
ICD	3 (3.0%)
CRT	16 (15.8%)

Data expressed as mean ± standard deviation, median (25th–75th percentiles), or absolute number (percentage)

*ACEI* angiotensin-converting enzyme inhibitor, *ARB* angiotensin receptor blocker, *CRT* cardiac resynchronization therapy, *ICD* implantable cardioverter device, *LVEF* left ventricular ejection fraction, *MRA* mineral corticoid receptor antagonist, *NYHA* New York Heart Association

^a^eGFR < 60 ml/min/1.73 m^2^

^b^Hb of < 12 g/dl in women and < 13 g/dl in men

**Fig. 1:**
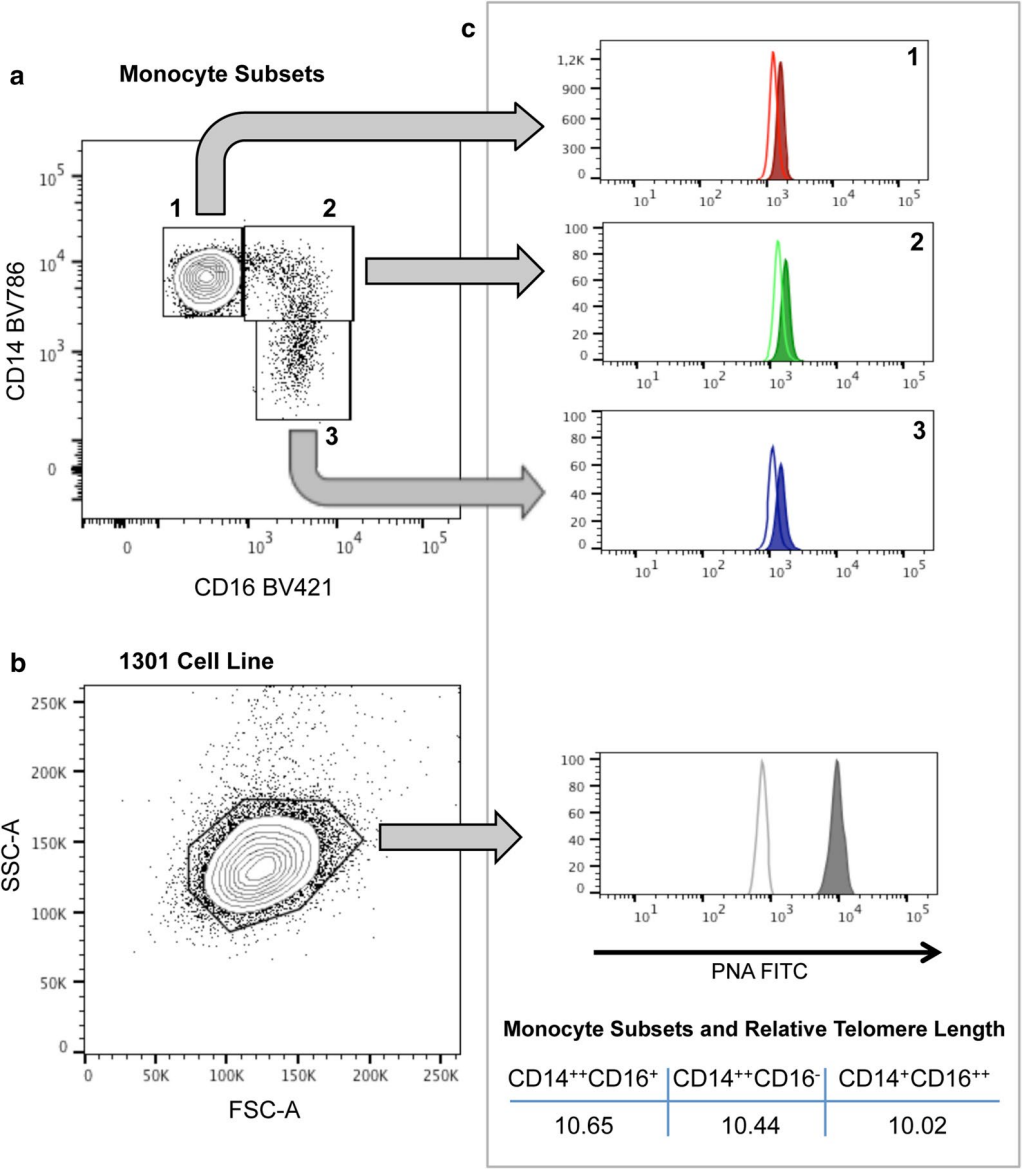
Representative flow-FISH analysis of TL in monocyte subsets from an ambulatory patient with HF. Cells were previously selected by G0/G1 DNA Content, scatter properties and CD86 and CD15 markers for monocytes and 1301 cell line selection as described in Materials and methods (data not shown). Monocyte subsets were analyzed based on their CD14 and CD16 expression (**a**: 1. CD14^++^CD16^−^; 2. CD14^++^CD16^+^; 3. CD14^+^CD16^+^). Scatter properties of 1301 cell line and gating was shown in **b**. Hybridization of FITC-labeled Telomere Probe (PNA; color filled histograms) and control (without probe; empty histograms) in the three existing monocyte subsets and internal reference control 1301 cell line **c**. The relative TL value for each monocyte subset was finally calculated as the ratio between the MFI of each subset and the MFI of the control cells. Corrections were also made for the DNA index of G0/G1 cells

**Fig. 2:**
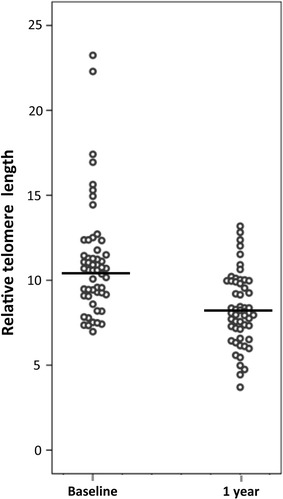
Scatter dot plot with mean line (horizontal bar) graphic of relative telomere lengths at baseline and at 1 year. N = 54

Within this cohort of HF patients, we measured three monocyte subsets—referred to as classical (CD14^++^CD16^−^), intermediate (CD14^++^CD16^+^), and nonclassical (CD14^+^CD16^++^)—along with their respective TLs. The coefficient of variation of the intra-assay for duplicates was 5.08% for monocyte population with minor differences between subsets. Across all runs, inter-assay coefficient of variation was 71.82% for unprobed and 22.81% for probed, similar to previously described measurements by other groups [14]. Figure 1 shows a representative TL analysis of a whole-blood sample from a patient with HF. Collectively, we found that the TL values were highly correlated between the different monocyte subsets: CD14^++^CD16^+^ vs. CD14^++^CD16^−^, R = 0.95, p < 0.001; CD14^++^CD16^+^ vs. CD14^+^CD16^++^, R = 0.90, p < 0.001; and CD14^++^CD16^−^ vs. CD14^+^CD16^++^, R = 0.89, p < 0.001. Table 2 shows TL at baseline and at the 1-year follow-up, as well as the percent change, for the whole-blood sample of monocytes and for each monocyte subset. Within the subgroup of patients for whom blood samples were available for both time-points, we found statistically significant ~ 22% attrition of mean monocyte TL (11.1 ± 3.3 vs. 8.3 ± 2.1, p < 0.001). Mean monocyte TL change was –22% ± 20 (Fig. 2 and Table 2). Monocyte TL reduction occurred in 96.3% of patients (ranging from − 3.1 to − 66.7%); in 28 of these patients (51.9%) monocyte TL reduction was ≥ − 20%. TL at baseline, TL at the 1-year follow-up, and the change in TL over 1 year did not significantly differ between the monocyte subsets (all p values > 0.1). In particular, there was no difference in TL attrition between genders (− 23.5 ± 14.4 and − 21.4 ± 22.5% in women and men, respectively). Furthermore no relationship between TL attrition and age (< 70 years vs. older), sex, ischemic etiology, presence of diabetes mellitus or HF duration (< 48 months vs. longer) was found.

**Table 2:** Telomere lengths at baseline and at 1 year

Baseline	n = 101
Monocytes, whole	10.3 ± 3.3
CD14^++^CD16^−^	10.5 ± 3.6
CD14^++^CD16^+^	10.1 ± 3.3
CD14^+^CD16^++^	10.3 ± 3.1

1 year	N = 54
Monocytes, whole	8.3 ± 2.1
CD14^++^CD16^−^	8.3 ± 2.2
CD14^++^CD16^+^	8.4 ± 2.3
CD14^+^CD16^++^	8.3 ± 2.1

%, Δ	N = 54
Monocytes, whole	− 22 ± 20
CD14^++^CD16^−^	− 25 ± 20
CD14^++^CD16^+^	− 19 ± 23
CD14^+^CD16^++^	− 21 ± 22

Data expressed as mean ± standard deviation

Mean monocyte TL was not significantly related to any demographic or clinical characteristics, except for the presence of atrial fibrillation (p = 0.01) while a tendency to inverse correlation was observed with age (p = 0.09) (Table 3). Over the mean follow-up of 2.3 ± 0.6 years, 17 patients died, 17 required hospital admission due to HF, and 29 suffered the composite end-point of death or HF hospitalization. Table 4 shows Cox regression analyses for all-cause death and for the composite end-point of all-cause death or HF hospitalization. These outcomes were not associated with baseline TL or with change in TL over 1 year.

**Table 3:** Baseline monocyte Telomere lengths according to demographic and clinical characteristics

	n = 101	p
Age (years)	R = − 0.17	0.09
Sex		0.44
Male	10.2 ± 2.9	
Female	10.7 ± 4.2	
Etiology		0.90^§^
Ischemic heart disease	10.4 ± 3.5	
Dilated cardiomyopathy	10.3 ± 4.4	
Hypertensive cardiomyopathy	10.5 ± 3.0	
Alcoholic cardiomyopathy	9.5 ± 1.8	
Valvular disease	9.1 ± 1.8	
Hypertrophic cardiomyopathy	11.3 ± 3.3	
Other	10.9 ± 2.5	
HF duration in months	Rho = − 0.13	0.20
LVEF	R = 0.14	0.18
NYHA functional class		0.85^§^
I	10.4 ± 3.5	
II	10.4 ± 3.6	
III	10.0 ± 3.3	
Hypertension		0.66
Yes	10.2 ± 3.5	
No	10.5 ± 2.3	
Diabetes mellitus		0.24
Yes	10.7 ± 3.4	
No	9.9 ± 3.1	
Renal failure^a^		0.31
Yes	10.0 ± 2.8	
No	10.6 ± 3.6	
Anemia^b^		0.23
Yes	10.8 ± 3.1	
No	10.0 ± 3.3	
Atrial fibrillation/flutter		0.01
Yes	9.3 ± 2.6	
No	11 ± 3.5	
Obesity		0.23
Yes	10.9 ± 3.6	
No	10.1 ± 3.1	
Smoker		0.48
No	10.4 ± 3.3	
Past	10.3 ± 3.3	
Current	8.6 ± 2.4	

Data expressed as mean ± standard deviation, median (25th–75th percentiles), or absolute number (percentage). R and Rho according to Pearson and Spearman correlation, respectively

*LVEF* left ventricular ejection fraction, *NYHA* New York Heart Association

^§^Scheffe post hoc analyses did not reveal any statistical difference between individual items

^a^eGFR < 60 ml/min/1.73 m^2^

^b^Hb of < 12 g/dl in women and < 13 g/dl in men

**Table 4:** Cox regression analysis for risk of all-cause death and the composite end-point of all-cause death or heart failure hospitalization based on Telomere length

	All-cause death			Composite endpoint		
	HR	[95% CI]	p value	HR	[95% CI]	p value
Monocytes, whole	1.02	[0.89–1.18]	0.76	1.00	[0.90–1.12]	0.97
CD14^++^CD16^−^	1.00	[0.87–1.13]	0.94	1.00	[0.91–1.11]	0.93
CD14^++^CD16^+^	1.03	[0.91–1.18]	0.63	1.02	[0.91–1.13]	0.78
CD14^+^CD16^++^	1.04	[0.90–1.20]	0.59	0.98	[0.97–1.11]	0.59
Monocytes, % ∆	0.99	[0.95–1.03]	0.57	0.98	[0.95–1.01]	0.24
CD14^++^CD16^−^, % ∆	1.00	[1.00–1.04]	0.81	0.98	[0.95–1.01]	0.12
CD14^++^CD16^+^, % ∆	0.99	[0.95–1.03]	0.51	0.98	[0.96–1.01]	0.21
CD14^+^CD16^++^, % ∆	0.98	[0.94–1.03]	0.44	1.00	[0.97–1.03]	0.75

% ∆ available in 54 patients

## Discussion

The results of this longitudinal study revealed that TL significantly declined (by ~ 22%) within a year in circulating monocytes from patients with HF. Our data did not indicate that TL was correlated with various monocyte subsets or with HF outcomes. To our knowledge, this is the first study to describe the course of telomere length change within a well-characterized cohort of patients with HF using simultaneous flow-FISH.

Telomere biology is linked to aging and age-associated pathologies, and preliminary data from cross-sectional studies reveal shorter telomeres in HF patients compared to healthy age- and gender-balanced controls, based on measurements in circulating leukocytes using a conventional qPCR method [5]. Moreover, TL is reportedly associated with the severity of HF symptoms and outcome [5, 15], and with worse renal function in subjects with HF, which is a powerful predictor of outcome [16, 17]. While TL is usually measured in leukocytes, it has also been evaluated in cardiac tissue from patients with HF [18]. There remains a need for large, prospective, longitudinal studies to acquire more in-depth insights into the relationship between TL and HF [2]. Herein, we used a novel flow-FISH method to specifically determine TL attrition in circulating monocyte subsets [8].

All previous studies of TL in HF have been cross-sectional in design, and thus have not provided information about possible changes in TL over time or whether any such changes are related to outcomes. Our current report provides the first evidence of accelerated telomere erosion in the monocytes of patients with HF over 1 year. In our study, 96.3% of patients with HF showed shortening of TL at the 1-year follow-up. While this proportion is similar to that observed in general population studies [19], the rate of decline in our cohort is significantly higher than in normal individuals. Indeed, over 50% of the patients exhibited a telomere attrition exceeding − 20% at the 1-year follow-up. The MRC National Survey of Health and Development (NSHD, also known as the 1946 British Birth Cohort) exquisitely reported longitudinal measures of telomere length in a large cohort comprising mainly cardiovascular disease-free participants. In this cohort of healthy individuals, telomere length shortening over ten years was ~ − 2%, as measured by real-time PCR [19].

It is beyond the scope of this study to determine exactly why telomeres shorten faster in patients with HF. However, oxidative stress and inflammation are considered the most important factors contributing to telomeric DNA loss [6], which in turn may favor the development of structural tissue damage. It is postulated that shorter TL may be an irremediable intracellular mechanism facilitating or even causing HF. Indeed, experimental data suggest that telomere shortening partly mediates apoptosis in HF [18]. Furthermore, telomere attrition may lead to increased levels of dysfunctional senescent cells (e.g., circulating monocytes) in tissues and organs, potentially explaining the lower threshold for expressing clinical manifestation of disease [2]. A biomarker’s value is largely determined by its capacity to reflect prognosis or change in disease progression; therefore, we followed patients for a mean of 2.3 years. Our data showed no correlation between the rate of telomere attrition and the primary endpoints, suggesting that telomere shortening rate may not be a clinically useful measurement or a viable surrogate marker of disease progression. On the other hand, it is possible that a longer observation period or a larger sample may be required to evaluate these clinical endpoints.

The presented findings also revealed that monocyte TL was associated with atrial fibrillation. Recent investigations of the relationship between TL and atrial fibrillation have produced controversial findings [20]. In the Cardiovascular Health Study (CHS), Roberts et al. [21] compared patients with and without atrial fibrillation, and found no relationship between mean TL and atrial fibrillation. In contrast, Carlquist et al. [22] reported shorter TL in patients with atrial fibrillation, both before and after adjustment for age and other cardiovascular risk factors. However, the exact mechanism leading to shorter TL in patients with atrial fibrillation remains elusive.

Of note, since participants were drawn from the general population that visited a structured HF Clinic located within a tertiary university hospital and our cohort included mainly male patients with ischemic heart disease, further research is needed to determine whether our findings can be generalized to other HF cohorts, such as patients with HF and preserved ejection fraction. As limitations, our sample size or duration of observation (2.3 years) seem to be insufficient to show significant associations between telomere shortening and the tested outcomes. We thus hypothesize that larger follow-up period and many measurement time-points will be crucial to further validate our findings. However, to implement a biomarker in precision medicine, it must show clinical applicability in small numbers of patients, if not in individual subjects.

## Conclusions

In summary, the present longitudinal observational study revealed a 22% reduction in TL over 1 year in monocytes from ambulatory patients with HF. Baseline TL and change in TL were not significantly associated with outcomes; therefore, the change in TL is not likely to be a useful biomarker of HF progression.

## Additional file

**Additional file 1: Table S1.** Baseline Characteristics of the Study Participants with one year sample.

## Abbreviations

DNA: deoxyribonucleic acid
FITC: fluorescein isothiocyanate
Flow-FISH: flow cytometry-fluorescence in situ hybridization
HF: heart failure
MFI: median fluorescence intensity
PNA: peptide nucleic acid
qPCR: quantitative polymerase chain reaction
TL: telomere length
