# Supplementary material for: Telomere attrition in heart failure: a flow-FISH longitudinal analysis of circulating monocytes
Source: J Transl Med. 2018 Feb 20;16:35. doi: 10.1186/s12967-018-1412-z (PMC5819711; doi:10.1186/s12967-018-1412-z)
Supplement: Supplementary file 1 — Additional file 1: Table S1. Baseline Characteristics of the Study Participants with one year sample. [file 12967_2018_1412_MOESM1_ESM.docx]

**TABLE S1** Baseline Characteristics of the Study Participants with one year sample

|  | **n = 54** |
| --- | --- |
| **Age (years)** | 65.6 ± 12.1 |
| **Male sex** | 39 (72.2%) |
| **Etiology** |  |
| Ischemic heart disease | 25 (46.3%) |
| Dilated cardiomyopathy | 9 (16.7%) |
| Hypertensive cardiomyopathy | 7 (13.0%) |
| Alcoholic cardiomyopathy | 5 (9.3%) |
| Valvular disease | 2 (3.7%) |
| Hypertrophic cardiomyopathy | 1 (1.9%) |
| Other | 5 (9.3%) |
| **HF duration in months** | 41.0 (13.2–85.1) |
| **LVEF** | 41.89% ± 11.5 |
| **NYHA functional class** |  |
| I | 5 (9.3%) |
| II | 40 (74.1%) |
| III | 9 (16.7%) |
| **Obesity** | 15 (27.8 %) |
| **Smoker** |  |
| Current | 2 (3.7%) |
| Past | 32 (59.3%) |
| **Co-morbidities** |  |
| Hypertension | 37 (68.5%) |
| Diabetes mellitus | 26 (48.1%) |
| Renal failure* | 21 (38.9%) |
| Anemia^#^ | 22 (40.7%) |
| Atrial fibrillation/flutter | 20 (37.0%) |
| **Treatments** |  |
| ACEI/ARB | 48 (88.9%) |
| Beta-blockers | 51 (94.4%) |
| MRA | 32 (59.3%) |
| Loop diuretics | 41 (75.9%) |
| Digoxin | 8 (14.8%) |
| Ivabradine | 8 (14.8%) |
| Statins | 46 (85.2%) |
| ICD | 11 (20.4%) |
| CRT | 10 (18.5%) |

Data expressed as mean ± standard deviation, median (25th–75th percentiles), or absolute number (percentage).*eGFR < 60 ml/min/1.73 m^2^; ^#^Hb of <12 g/dl in women and <13 g/dl in men. ACEI = angiotensin-converting enzyme inhibitor; ARB = angiotensin receptor blocker; CRT = cardiac resynchronization therapy; ICD = implantable cardioverter device; LVEF = left ventricular ejection fraction; MRA = mineral corticoid receptor antagonist; NYHA = New York Heart Association.
